# The effect of socioeconomic disadvantage on prescription of guideline-recommended medications for patients with acute coronary syndrome: systematic review and meta-analysis

**DOI:** 10.1186/s12939-017-0658-z

**Published:** 2017-08-31

**Authors:** Karice K. Hyun, David Brieger, Mark Woodward, Sarah Richtering, Julie Redfern

**Affiliations:** 1 Sydney Medical School, University of Sydney, Sydney, Australia; 2 The George Institute for Global Health, Sydney, Australia; 3 Department of Cardiology, Concord Hospital, University of Sydney, Sydney, Australia; 4 The George Institute for Global Health, Nuffield Department of Population Health, University of Oxford, Oxford, UK; 5 Geneva University Hospital, Geneva, Switzerland; 6 Level 10, King George V Building, 83-117 Missenden Rd, Camperdown, NSW 2050 Australia

**Keywords:** Acute coronary syndrome, Socioeconomic status, Medication prescription, In-hospital care, Health inequity, Systematic review

## Abstract

**Background:**

There are varying data on whether socioeconomic status (SES) affects the treatment in patients with acute coronary syndrome (ACS). Our aim was to obtain a reliable estimate of the effect of SES on discharge prescription of medications following an ACS through systematic review and meta-analysis.

**Methods:**

Medline, EMBASE and Global Health were searched systematically on 6th April 2016. Studies were eligible if the participants had ACS and reported the rate/odds of guideline-recommended ACS medications prescription (aspirin, antiplatelet, beta blocker, angiotensin co-enzyme inhibitors (ACEi)/angiotensin receptor blockers (ARB) and statin) at discharge stratified by SES. A meta-analysis was performed to pool the estimates, comparing the prescription ratio (PR) between the lowest and the highest SES groups.

**Results:**

Of 252 articles found from the search, seven met the eligibility criteria and it included 41,462 (20,986 from the lowest SES group) patients. We found that the individual/neighbourhood level SES did not affect the prescription of aspirin (PR (95% CI): 0.97 (0.91, 1.03)), but for beta blocker and statin, the lowest SES group were disadvantaged (0.84 (0.73, 0.94), 0.80 (0.62, 0.98), respectively). In contrast, ACEi were prescribed more often to the lowest individual/neighbourhood level SES group than the highest (1.13 (1.05, 1.22)). Although the risk of bias was low, there was considerable heterogeneity between the studies.

**Conclusions:**

Despite the recommendations to close the treatment gap, the rate of prescription of guideline-recommended medications in managing ACS is significantly different between patients with the lowest and the highest groups. A solution is needed to provide equitable care across the SES groups.

**PROSPERO Registry:**

Systematic review registration no.: CRD42016048503. Registered 28 September 2016.

## Introduction

Acute coronary syndrome (ACS) is a high-risk form of coronary atherosclerosis, consisting of myocardial infarction (MI) and unstable angina (UA). About 1.4 million people are hospitalised with a diagnosis of ACS every year in the United States [1]; in Australia, more than 95,000 are hospitalised each year with a readmission rate within 6 months of about 20% [2, 3]. To reduce the risk of recurrence or death, recommended treatment guidelines for optimal treatment are in place [4–7]. An important part of these guidelines is the prescription of recommended medications at discharge from hospital. The recommended medications include aspirin and antiplatelet, which prevent blood to clot; beta blocker and angiotensin co-enzyme inhibitor (ACEi)/angiotensin receptor blocker (ARB), which are blood pressure lowering medications; and statin/lipid lowering therapy, which are cholesterol lowering medications [8, 9]. These evidence-based medications have been proven to reduce the risk of mortality and major adverse cardiovascular events, with further risk reduction when used in combination, and are recommended to be taken long-term [4–7, 10, 11]. However, studies suggest that there are social subgroups that receive optimal treatment less often [12–16].

World Health Organisation (WHO) reports that social factors such as socioeconomic status (SES) can influence patients’ treatment of illness as well as health [17]. Previous studies found that patients with low SES have significantly higher risk of an outcome or death due to a chronic disease, including chronic obstructive pulmonary disease outcomes, cardiovascular disease [18–20]. Similar results were found for ACS, where the lowest SES group had the highest rates of MI and death due to coronary heart disease [21]. When it seems appropriate for those with ACS and low SES to be treated more rigorously compared to those with high SES, some studies have reported that patients in the lowest SES group were disadvantaged in receiving some guideline recommended discharge medication prescriptions [12–16]. However, there were also evidence suggesting that patients receive comparable rate of prescription across the SES groups [13–15, 22, 23].

Therefore, to better understand whether the treatment gap exists between the SES groups in patients with ACS, we aimed to synthesise results from studies that reported on the effect of SES on discharge prescription of medications for patients with ACS.

## Methods

Pre-specified methods for this systematic review were registered in the International prospective register of systematic reviews (PROSPERO) Registry (registration no.: CRD 42016048503). Studies were eligible for review if the participants were patients with a diagnosis of ACS, stratified by patients’ SES and the studies reported the rate or the odds of any of the prescription of guideline-recommended ACS medications (aspirin, antiplatelet, ACEi/ARB, beta blocker and/or statin/lipid-lowering therapy) at hospital discharge. Due to the nature of the question, only observational studies were included. There were no language or date restrictions. The Preferred Reporting Items for Systematic Reviews and Meta-analyses (PRISMA) guidelines were used to report this systematic review [24].

### Search strategy/data extraction

The search strategy was conducted after consultation with librarians. The databases searched for this review were MEDLINE (1946 - 6th April 2016), EMBASE (1974 - 6th April 2016) and Global Health (1973 - 6th April 2016), via Ovid. Search terms related to ACS, SES (employment, occupation, income and education) and recommended medications (aspirin, other antiplatelet, beta blocker, ACE/ARB, and statin/lipid-lowering therapy) were used (Appendix 1) to find all studies that compared the prescription of recommended medication for ACS patients by SES. Additional articles were obtained through manual search of reference lists. Duplicates were removed using a reference management system (Endnote X7) and manual assessment. All remaining studies were screened initially by reading the titles and abstracts, and then, the relevant studies were selected and the full texts of the studies were read to determine if they met the inclusion criteria. The literature search was completed by one author (KH) and validated by a second author (SR). These two authors performed study selection independently, and any disagreement was resolved through discussion.

Data were extracted by one author (KH) and check by a second author (SR) for accuracy. Adjusted or unadjusted prevalence ratios and rates of medication prescription by SES groups were extracted. Other extracted data included study details (authors’ name, aim, year of recruitment, country of recruitment), ACS diagnosis (MI or ACS), the definition of SES, the number of SES groups compared, prescribed medications.

### Risk of bias

The Newcastle-Ottawa Scale was used to assess the risk of bias of the observational studies for the review and the meta-analyses [25]. The assessment was performed independently by two authors (KH, SR). The components assessed were Selection (four items of assessment), Comparability (one item) and Outcome (three items), where a study can be awarded a maximum of one star for each item of assessment within the Selection and Outcome components, and a maximum of two starts for Comparability.

### Outcomes

Outcomes of interest were the prescription of recommended medications for ACS management, which were aspirin, other antiplatelet, beta blocker, ACEi/ARB, and statin/other lipid-lowering medications, separately and combined. Varying definitions of SES were accepted if the measure of SES was credible and categorised. Regardless of the number of SES categories reported, only the lowest and the highest groups were compared for this review. The reason for this was that the studies included in this review reported between two and four SES groups, where three of the seven studies only reported two SES groups. For the four studies that reported on more than two groups of SES, the prescription rate across all SES groups have been included in the Appendix (Appendix 2).

For studies that compared the prescription of medication using more than one definition of SES, the most frequently used measure of SES among the other studies was selected to reduce the diversity of the SES definitions when pooling the studies.

### Statistical analysis

A priori, the outcome for this study was defined as the pooled prescription ratio comparing the lowest (most deprived) and highest (least deprived) individual/neighbourhood level SES groups of those groups reported in each study. Sensitivity analyses was performed by including the results from comparing between country level SES. For the studies that reported the rate of recommended medication prescription only, the prescription ratio (PR) and corresponding 95% confidence interval (CI) for the prescription of recommended medication between the lowest and the highest SES groups were estimated from the rate of prescription and the sample size, for each study using the following formulae:

$$ \mathrm{Prescription}\  \mathrm{ratio}=\frac{p_1}{p_2} $$

Where p_1_ = proportion of patients in the lowest SES group with a prescription of medication; and.

p_2_ = proportion of patients in the highest SES group with a prescription of medication.

$$ 95\%\mathrm{confidence}\  \mathrm{interval}={e}^{\ln \left(\widehat{PR}\right)\pm 1.96\sqrt{\frac{\left({n}_1-{x}_1\right)/{x}_1}{n_1}+\frac{\left({n}_2-{x}_2\right)/{x}_2}{n_2}}} $$

Where n_1_ = total patients in the lowest SES group.

n_2_ = total patients in the highest SES group.

x_1_ = number of patients in the lowest SES group with a prescription of medication.

x_2_ = number of patients in the highest SES group with a prescription of medication.

Meta-analysis was performed using the random effects model to pool the estimates. For medications that had been examined in more than two studies, I^2^ was used to quantify heterogeneity, and Cochran’s Q test to test the heterogeneity (*p*-value of <0.1 will be considered statistically significant for the Q test). Publication bias was tested using the funnel plot and Egger’s test.

## Results

**Fig. 1:**
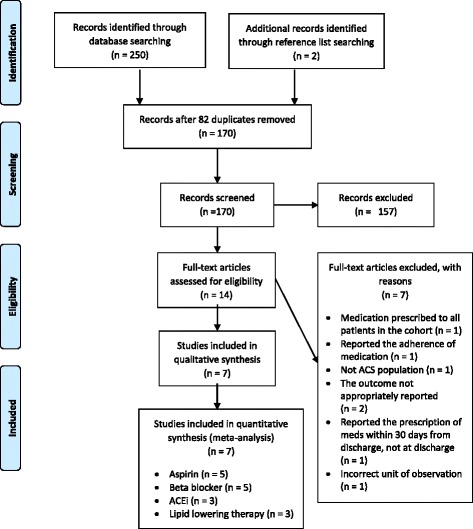
Flowchart of study selection. ACS: acute coronary syndrome; ACEi: angiotensin co-enzyme inhibitors

Of 252 articles found from the initial search, 14 articles were assessed in full-text and seven articles met the eligibility criteria to be included in the systematic review (Fig. 1). The measures of SES included income, education, deprivation score and country income level (Table 1). Characteristics of the seven articles are shown in Table 1. Two of the studies analysed different medications from one dataset [12, 22]. One study focused on international variations, and countries were compared according to SES; in this instance, the outcomes for the country with the low/middle SES was compared to that with the high as stratified in the original article [18]. Four studies focused on population subgroups within regions and compared neighbourhoods within these regions [12, 14, 22, 23]. Two studies reported patient level SES [13, 16]. In total, the seven articles included ACS 41,462 patients in the lowest and highest SES groups together, of which 20,986 were from the lowest group. Of the total, 392 patients were stratified by country level income, and 127 of those were from the lowest income countries [15]. In regards to the outcomes reported, five articles examined the prescription of aspirin [12–15, 23], five articles on beta blocker [12–15, 23], three articles on ACE [12, 13, 15], three articles on lipid lowering therapy [15, 16, 22] and two articles on combination of medications [12, 15].

**Table 1:** Study characteristics

First author, year	Country	Year of data collection	Analysed sample size (Lowest + highest SES group) (% lowest SES)	ACS diagnosis	SES type (groups reported in the original articles)	Medication class	Adjustments
Barakat et al., 2001 [23]	The United Kingdom	1988–1996	593 (49)	Myocardial infarction	Neighbourhood deprivation score (Quartiles 1 to 4)	Aspirin and beta blocker	No adjustments
Foraker et al., 2010 [12]	The United States	1993–2002	8596 (52)	Definite or probable myocardial infarction	Neighbourhood household income (low, medium and high)	Aspirin, beta blocker and ACEi	Medicaid status, race, gender, age, study community, year of myocardial infarction, hospital type (teaching vs. non-teaching), current or past history of hypertension, diabetes or heart failure and presence of cardiac pain.
Gerber et al., 2008 [13]	Israel	1992–1993	1263 (47)	Myocardial infarction	Income (below average and average/above average)	Aspirin, beta blocker, ACEi	No adjustments
Kawecka-Jaszcz et al., 2003 [16]	Poland	1996–1999	758 (75)	Acute coronary syndrome, PCI and isolated CABG	Education (≤13 and >13 years)	Lipid-lowering therapy	No adjustments
Kitzmiller et al., 2013 [22]	The United States	1999–2002	3291 (49)	Definite or probable myocardial infarction	Neighbourhood income (low, medium and high)	Lipid-lowering therapy	Medicaid status, race, gender, age, study community, year of myocardial infarction, hospital type (teaching vs. non-teaching), current or past history of hypertension, diabetes or heart failure and presence of cardiac pain.
Rao et al., 2004 [14]	The United States	1994–1996	26,568 (50)	Myocardial infarction	Neighbourhood income (low, middle and high)	ACEi and beta blocker	No adjustments
Shimony et al., 2014 [15]	India, Pakistan, Tunisia, Canada and the United States	2005–2009	392 (32)	Acute coronary syndrome	Country income level (low/middle and high)	Aspirin, clopidogrel, beta blocker, statin, ACEi and ARB	No adjustments

*SES* socioeconomic status, *ACS* acute coronary syndrome, *PCI* percutaneous coronary intervention, *CABG* coronary artery bypass graft, *ACEi* angiotensin co-enzyme inhibitor, *ARB* angiotensin receptor blockers

### Aspirin prescription

**Fig. 2:**
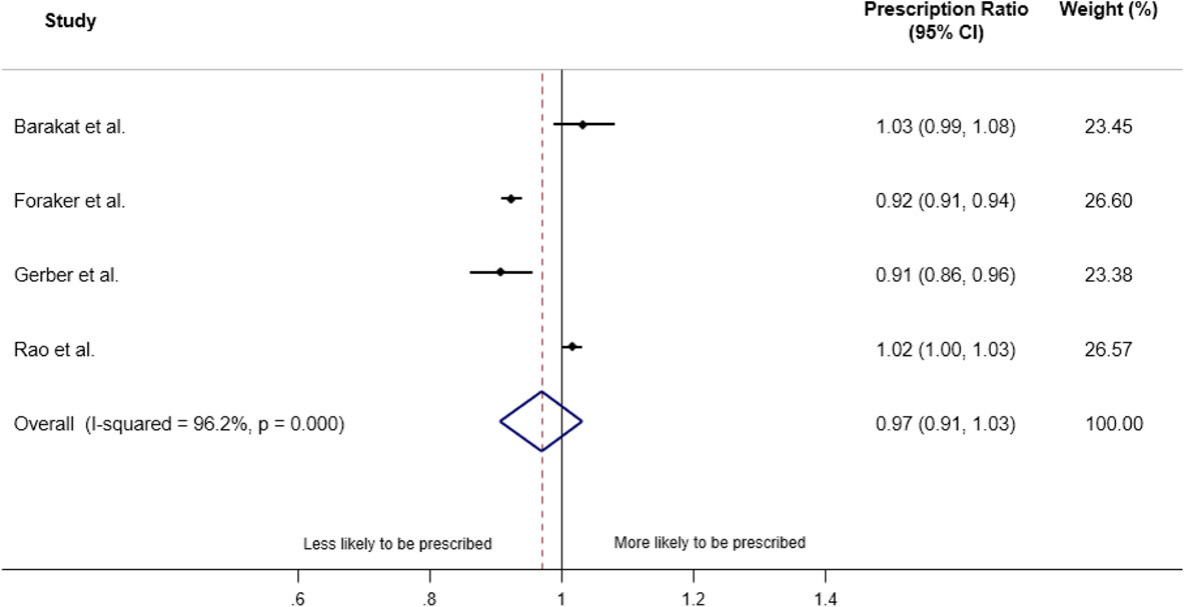
Meta-analysis of the effect of socioeconomic status (lowest vs. highest) on prescription of aspirin. CI: confidence interval

The four studies that reported the aspirin prescription at discharge by the individual/neighbourhood level SES groups included 36,427 (18,386 in the lowest SES group) patients [12–14, 23]. Overall, there was no significant difference in the likelihood of prescription between the lowest and the highest SES groups (PR (95% CI): 0.97 (0.91, 1.03)) (Fig. 2), however, considerable heterogeneity was observed (I^2^ = 96%, *p* = 0.0001). The PR (95% CI) between the country level SES was 1.01 (0.98, 1.05) [15]. The sensitivity analysis, which pooled the results comparing the individual/neighbourhood and the country level SES, also showed no difference in the receipt of aspirin between the SES groups (0.98 (0.93, 1.03)).

### Beta blocker prescription

**Fig. 3:**
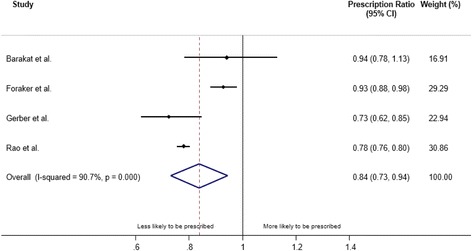
Meta-analysis of the effect of socioeconomic status (lowest vs. highest) on prescription of beta blocker. CI: confidence interval

The lowest SES group were less likely to receive the prescription of beta blocker at discharge than the highest SES group. Beta blocker was explored in the same four studies comparing the individual/neighbourhood level SES that reported the prescription of aspirin [12–14, 23]. The pooled PR for the prescription of beta blocker at discharge was 0.84 (95% CI: 0.73, 0.94) (Fig. 3) but the I^2^ statistic was 91%, indicating substantial heterogeneity between the studies (*p* = 0.0001). The PR (95% CI) comparing the lowest and the highest country level SES was 0.93 (0.85, 1.02) [15]. The pooled PR was similar even after including the country level SES (0.86 (0.76, 0.95)).

### Lipid-lowering therapy prescription

The total number of patients included in the two studies that compared between the individual/neighbourhood level SES and reported lipid lowering therapy prescription at discharge was 4049, and of these, 2183 were in the lowest SES group [16, 22]. Both papers showed that patients in the lowest SES group were less likely to receive the prescription compared to the highest group, and the pooled PR was 0.80 (95% CI: 0.62, 0.98). The PR (95% CI) between the country level SES was 0.85 (0.77, 0.94) [15]. The sensitivity analysis also showed that patients in the lowest SES group were less likely to receive the prescription of lipid lowering medication compared to those in the highest SES group (0.82 (0.72, 0.92)).

### ACEi prescription

ACEi prescription at discharge was reported in two studies (9859 patients, and 5032 patients from the lowest SES) [12, 13]. In contrast to the other medications, the lowest SES group were more likely to receive the prescription of ACEi than the highest SES group (PR (95% CI): 1.13 (1.05, 1.22)). Similar results were found when the country level SES groups were compared, but the PR was greater between the lowest and the highest SES groups (1.36 (1.21, 1.52)) [15]. Therefore, the PR from the sensitivity analysis was moderately greater (1.21 (1.05, 1.38)).

### Composite medications

Two studies compared the prescription of composite medications. One study compared the neighbourhood level SES and defined composite medications as the prescription of two or more of aspirin, beta blocker and ACEi [12], and the other study compared the country level SES and defined it as the prescription of aspirin, clopidogrel, statin and beta blocker [15]. The two studies found discordant results: the first study found no difference in the prescription of medications by SES whereas the second study reported that patients in the lowest SES group have 70% lower odds of receiving the prescription of medications compared to the highest SES group (odds ratio (95% CI): 0.3 (0.2, 0.6)).

### Risk of bias

Overall, the risk of bias was low. Most articles clearly reported the cohort selection, data collection, comparability of cohorts and assessment of outcome (Table 2). All studies obtained exposures from either medical records or a structured interview, and all studies, except one, reported to have collected the outcome data from the medical records. However, five of the seven articles did not adjust for confounders because finding the association between the SES groups and prescription of recommended ACS medications were not the primary aim of their study, increasing the risk of bias. Two criteria in the Outcome section regarding the follow-up were irrelevant for the objective of the current study, therefore, were not included in the assessment (Table 2). Also, there was no indication of publication bias (aspirin: *p* = 0.910; beta-blocker: *p* = 0.468; statin: *p* = 0.393; and ACEi: *p* = 0.827).

**Table 2:** Risk of bias

First author, year	Selection (out of 5)	Comparability (out of 2)	Outcome (out of 1)
Barakat et al., 2001 [23]	****	*	- -
Foraker et al., 2010 [12]	****	**	* - -
Gerber et al., 2008 [13]	****	*	* - -
Kawecka-Jaszcz et al., 2003 [16]	****	*	* - -
Kitzmiller et al., 2013 [22]	****	**	* - -
Rao et al., 2004 [14]	****	*	* - -
Shimony et al., 2014 [15]	****	*	* - -

Newcastle-Ottawa quality assessment scale

Two criteria regarding the follow-up were irrelevant for the objective of the current study, therefore, were not included in the assessment (denoted as “-”).

## Discussion

The current systematic review has assessed and analysed seven articles that compared the likelihood of prescription of guideline-recommended medications (aspirin, beta blocker, lipid-lowering therapy and ACEi) to ACS patients between the SES groups at hospital discharge. We found that individual/neighbourhood level SES had an effect on the prescription of beta blocker, statin and ACEi, but not aspirin. Beta blocker and statin were 16% and 20% less likely, respectively, to be prescribed to the lowest SES group than to the highest SES group. In contrast, ACEi was 13% more likely to be prescribed to patients in the lowest SES group compared to those in the highest SES group. The study that compared between the country level SES showed similar results, where the lowest SES group were less often prescribed beta blocker and lipid lowering therapy but more often prescribed ACEi than the highest SES group [15].

The opposing effect of SES on two blood pressure lowering medications (ACEi and beta blocker) was an interesting finding given that both medications are indicated for patients with hypertension and heart failure. It is widely known that ACS risk factors, including hypertension, heart failure and diabetes, are more prevalent in patients with low SES [26, 27], which was also found in the studies included in this review. Although ACEi and beta blocker are both similarly effective in controlling blood pressure [28], ACEi is recommended for patients with hypertension, diabetes and kidney disease, whereas, the use of beta blockers has been discouraged in patients with diabetes due to the masking of hypoglycaemic symptoms [29]. The increased prevalence of comorbidities in lower SES groups may be one of the reasons why ACEi was more often prescribed to the lowest than the highest SES group.

Three of the seven studies included in this review have explored the effect of a second socioeconomic measure, which also showed that patients with lower SES tended to receive prescriptions to recommended medication less often compared to those with higher SES. Gerber et al. reported a significant proportional difference in the percentage prescribed beta blockers between those with <12 years (32%) and ≥12 years (38%) of education [13]. However, the prescriptions of aspirin and ACEi were comparable (80% vs. 83% and 22% vs. 20%, respectively). After adjustment, Foraker et al. found that patients with Medicaid (predominantly those with low income) were less likely to receive aspirin (OR (95% CI): 0.92 (0.87, 0.98)) and composite medications (0.94 (0.88, 1.00)), although the prescription of composite medications were marginally significant with the upper CI on 1.00 [12]. The likelihood of being prescribed beta blocker and ACEi were not statistically significant. Kawecka-Jaszcz et al. explored the effect of employment on the prescription of lipid-lowering therapy, and found that the proportion of those who were prescribed lipid-lowering medication was lower by 8% for the unemployed than for the employed, however, the difference was not significant (39% vs. 47%; *p* > 0.05) [16]. Although the percent difference between the SES groups varied slightly between the socioeconomic measure used in the meta-analyses and the second socioeconomic measure, the results were comparable between different measures of SES. Regardless of the type of the SES, the rate of aspirin, beta blocker and lipid-lowering therapy prescriptions were lower for patients in the lowest SES group, whereas, the rate of ACEi prescription was higher for patients in the highest SES group.

ACS, being a disease with a high incidence of recurrent adverse clinical events, has recommended guidelines and clinical pathways in place to help clinicians provide optimal treatment to every patient. However, despite these inclusive recommendations, applicable to all patients, it is unclear as to why variation in the prescription of guideline-recommended medications between the SES groups is often found in studies. Reasons might include physicians’ clinical management that varies to accommodate patients’ financial status to lessen their burden, as was reported to have been done in primary care [30]. Further investigation would be needed to find factors that cause the discrepancy in the prescription of recommended medication at discharge to be able to find ways to provide equitable care for all patients and prevent a secondary event.

There were limitations to this review. First, the reviewed studies used observational data, which may be subject to reporting bias. Second, we could only compare between the lowest and the highest SES groups in the meta-analysis as some studies reported only two SES groups, therefore could not use complete data from all studies. Third, the studies being observational, comparison of likelihoods of the outcome after adjusting for covariates would have been ideal, however, due to the limited number of studies that aimed to find the effect of SES on the prescription of medications, studies with unadjusted data have also been included in the meta-analysis. Fourth, also because of the small number of studies found for this systematic review, the definitions and types of SES (deprivation score, income and education) and the level of SES (individual, neighbourhood and country) varied across the studies. Although subgroup analysis for the different types of SES could not be done, the results from the studies that reported the effect of two different types of SES on prescription of medications suggests that the different types of SES may not affect the study results greatly. Fifth, the studies included for this review may be limited due to our refined search terms. Sixth, the power to test for asymmetry and heterogeneity was substantially low with only 2–4 studies for each meta-analysis [31, 32].

## Conclusion

Prescriptions of guideline-recommended secondary prevention medications at discharge varied according to the SES of patients. Patients from the lowest SES group were less often prescribed beta blocker and statin, but more often prescribed ACEi compared to those from the highest SES group. Adherence to guidelines and policies needs to be promoted to reduce the discrepancies between the lowest and the highest SES groups.

## Appendix 1

1. exp. Acute coronary syndrome/.

2. exp. myocardial infarction/.

3. exp. unstable angina/.

4. ACS.tw.

5. 1 or 2 or 3 or 4.

6. exp. patients/.

7. exp. hospital/.

8. 5 and 6 and 7.

9. Socioeconomic.tw.

10. socio?economic.tw.

11. SES.tw.

12. Socio economic.tw.

13. Employment.tw.

14. Education*.tw.

15. Educational status.tw.

16. Income*.tw.

17. Unemployment.tw.

18. Occupations.tw.

19. 9 or 10 or 11 or 12 or 13 or 14 or 15 or 16 or 17 or 18.

20. Aspirin.tw.

21. Antiplatelet.tw.

22. Clopidogrel.tw.

23. Ticagrelor.tw.

24. Prasugrel.tw.

25. Beta?blocker.tw.

26. Blood pressure lowering.tw.

27. BP-lowering.tw.

28. Statin.tw.

29. (Lipid adj3 lowering).tw.

30. ACE.tw.

31. ACEi.tw.

32. ACE-i.tw.

33. angiotensin converting enzyme inhibitor.tw.

34. arb.tw.

35. Angiotensin II receptor blockers.tw.

36. discharge*.tw.

37. 20 or 21 or 22 or 23 or 24 or 25 or 26 or 27 or 28 or 29 or 30 or 31 or 32 or 33 or 34 or 35.

38. 36 and 37.

39. 5 and 19 and 38

## Appendix 2

**Table 3:** Prescription of medication by socioeconomic groups for studies that reported more than two socioeconomic groups

Study	No of SES groups	Medication	Group 1 (%)	Group 2 (%)	Group 3 (%)	Group 4 (%)	*P*-value
Barakat et al.	4	Aspirin	91	271	267	272	0.2
		Beta blocker	136	119	103	122	>0.2
Foraker	3	Aspirin	83	79	80		NA
		Beta blocker	62	72	75		NA
		ACEi	56	47	46		NA
Kitzmiller^a^	3	Lipid lowering medication	0.89 (0.79, 1.01)	0.98 (0.91, 1.07)	Reference		
Rao	3	Aspirin	69.7	69.6	68.6		>0.01
		Beta blocker	33.3	38.5	42.7		<0.01

*SES* socioeconomic status, *ACEi* angiotensin co-enzyme inhibitor

Group 1 refers to the most disadvantaged SES group and Group 3/4 refers to the least disadvantaged SES group

^**a**^Reported as prevalence ratio (95% confidence interval)

## Abbreviations

ACEi: Angiotensin co-enzyme inhibitors
ACS: Acute coronary syndrome
ARB: Angiotensin receptor blockers
CI: Confidence interval
MI: Myocardial infarction
PR: Prescription ratio
SES: Socioeconomic status
UA: Unstable angina
